# Description and evaluation of a national humanitarian opioid poisoning education and naloxone distribution program

**DOI:** 10.17269/s41997-025-01027-3

**Published:** 2025-04-16

**Authors:** Bruna dos Santos, Alexandra Kubica, Anna Maria Subic, Nick Rondinelli, Ben Evans-Durán, Melina Hanna, Don Marentette, Joanna Muise, Kevin Paes, Meghan Riley, Samiya Bhuiya, Jeannene Crosby, Keely McBride, Joe Salter, Aaron M. Orkin

**Affiliations:** 1 Dalla Lana School of Public Health, University of Toronto, Toronto, ON Canada; 2 Heart to Heart First Aid CPR Services Inc., Toronto, ON Canada; 3 Action First Aid, Barrie, ON Canada; 4 The Canadian Red Cross Society, Ottawa, ON Canada; 5 Canadian Red Cross Opioid Harm Reduction Advisory Council, Ottawa, ON Canada; 6 Department of Family and Community Medicine, University of Toronto, Toronto, ON Canada; 7 Li Ka Shing Knowledge Institute, Unity Health Toronto, Toronto, ON Canada; 8 Department of Emergency Medicine, Unity Health Toronto, Toronto, ON Canada

**Keywords:** Opioids, Opioid overdose education, Naloxone distribution, Harm reduction, Program evaluation, First aid, Opioïdes, Sensibilisation aux surdoses d’opioïdes, Distribution de naloxone, Réduction des dommages, Évaluation de programme, Premiers secours

## Abstract

**Setting:**

Canada’s opioid poisoning crisis claimed 49,105 lives from January 2016 to June 2024. Opioid poisoning education and naloxone distribution programs can reduce fatalities, although access remains inconsistent across Canada. These programs have mostly been delivered in person through community, healthcare, and social service agencies.

**Intervention:**

The Canadian Red Cross implemented a national, free, bilingual, virtually accessible, opioid harm reduction program, leveraging its experience in first aid education and community relationships as a humanitarian organization. The Opioid Harm Reduction program launched three new courses and added opioid poisoning content to four existing courses. Courses were adapted continually based on the feedback of people with lived experience of drug use and program participants. The program was delivered from January 2021 to March 2024 and evaluated through quantitative and qualitative methods.

**Outcomes:**

The program delivered 1,386,995 trainings and successfully reached diverse groups, including those from Indigenous (5.3%) and rural (25.2%) communities, but had an underrepresentation of men (34.3%) and individuals working in the construction industry (4.8%). Participants’ self-reported knowledge and confidence in responding to opioid poisoning increased across all courses (*p* < 0.001), particularly for learners without prior training. In total, 24,098 intranasal naloxone kits were distributed, 60.4% to Ontario, Manitoba, and British Columbia. Most participant feedback (82%) received was positive, highlighting the course’s simplicity and focus on stigma.

**Implications:**

The Canadian Red Cross Opioid Harm Reduction program advanced harm reduction, increased awareness of opioid poisonings, and situated the response to the opioid poisoning crisis as a community health effort.

## Résumé

**Lieu:**

La crise d’intoxication aux opioïdes au Canada a fait 49,105 victimes entre janvier 2016 et juin 2024. Les programmes de sensibilisation à l’intoxication aux opioïdes et de distribution de naloxone peuvent réduire les décès, mais l’accès à ces programmes varie encore d’un endroit à l’autre du pays. La plupart sont donnés en personne par des organismes de proximité et des organismes de soins de santé et de services sociaux.

**Intervention:**

Pour réduire les méfaits associés aux opioïdes, la Croix-Rouge canadienne a mis en œuvre un programme national gratuit, bilingue et virtuellement accessible en s’appuyant sur son expérience en formation aux premiers secours et en relations communautaires qui s’inscrit dans sa mission humanitaire. Dans le cadre de ce programme, elle a lancé trois nouveaux cours et ajouté du contenu sur l’intoxication aux opioïdes à quatre cours existants. Ces cours sont constamment adaptés en fonction des commentaires des participants et de personnes ayant une expérience vécue de l’usage de drogues. Le programme a été donné de janvier 2021 à mars 2024, puis évalué par des méthodes quantitatives et qualitatives.

**Résultats:**

Le programme a formé 1,386,995 personnes et a réussi à joindre des groupes divers, notamment dans les communautés autochtones (5,3%) et rurales (25,2%), mais les hommes (34,3%) et les personnes travaillant dans l’industrie du bâtiment (4,8%) y étaient sous-représentés. Les connaissances autodéclarées des personnes participantes et leur assurance de pouvoir intervenir en cas d’intoxication aux opioïdes se sont améliorées dans tous les cours (*p* < 0,001), surtout chez les personnes sans formation préalable. En tout, 24,098 trousses de naloxone intranasale ont été distribuées, 60,4% en Ontario, au Manitoba et en Colombie-Britannique. La plupart des personnes participantes (82%) ont laissé des commentaires positifs, en soulignant la simplicité des cours et leur accent sur la lutte contre la stigmatisation.

**Conséquences:**

Le programme de réduction des méfaits associés aux opioïdes de la Croix-Rouge canadienne a fait progresser la réduction des méfaits, accru la sensibilisation à l’intoxication aux opioïdes et positionné l’intervention contre cette crise comme un effort de santé communautaire.

## Introduction

The opioid poisoning crisis continues to severely impact Canadians, with 21 deaths per day reported in the first half of 2024, a majority (84%) occurring in British Columbia, Alberta, and Ontario (Federal, provincial, and territorial Special Advisory Committee on Toxic Drug Poisonings, 2024). Systematic reviews have identified initiatives that distribute naloxone and train individuals in its use as a suitable first aid intervention for lay responders, which can reduce opioid-related mortality (McDonald & Strang, 2016; Orkin et al., 2021; Razaghizad et al., 2021). Typically, opioid poisoning education and naloxone distribution programs are administered in-person by healthcare and community-based organizations in urban centres (Clark et al., 2014; Kesich et al., 2023; Razaghizad et al., 2021). Thus, ample opportunity exists to expand the administration and reach of these programs.

Humanitarian agencies are uniquely positioned to respond to the opioid poisoning crisis through their broad reach and community-based initiatives. Existing collaborations between communities and humanitarian agencies may serve as an avenue to destigmatize and promote harm reduction interventions as a mainstream response to a public health crisis. Funded by Health Canada and launched in 2021, the Canadian Red Cross Opioid Harm Reduction program aimed to expand the reach of opioid poisoning education and naloxone distribution in Canada, focusing on underserved communities. This article describes and presents evaluation findings of the Canadian Red Cross Opioid Harm Reduction program.

## Background

“Opioid overdose” is commonly used to describe the situation when an individual experiences toxicological complications from opioid use, leading to slowed breathing, unconsciousness, or death (Health Canada, 2017). In this paper, we use the term “opioid poisoning” to emphasize the toxicological emergency perpetuated by factors, such as the toxic drug supply and drug laws (Hansen et al., 2022), rather than excessive opioid dosage.

Opioid poisoning education and naloxone distribution programs can begin to address the impacts of the opioid poisoning crisis. Opioid poisoning education increases knowledge and confidence in recognizing and responding to opioid poisoning, and improves attitudes towards naloxone (Clark et al., 2014; dos Santos et al., 2024; Giglio et al., 2015; Pellegrino et al., 2021; Razaghizad et al., 2021). These programs can reduce fatalities by increasing the likelihood of individuals carrying naloxone and effectively using it in opioid poisoning emergencies (Cochrane Drugs and Alcohol Group et al., 2015; Razaghizad et al., 2021). Additionally, addressing the stigma and stereotypes surrounding naloxone, drug use, and opioid poisoning can reduce fatalities by increasing public awareness and acceptance of naloxone, and encouraging safer practices among those who use drugs (Knaak et al., 2019). Virtual programs can expand access to education and resources in underserved communities, including rural and remote areas (Bardwell & Lappalainen, 2021; dos Santos et al., 2024; Kesich et al., 2023).

## The Canadian Red Cross Opioid Harm Reduction program

The Canadian Red Cross (CRC), founded in 1909, is a not-for-profit corporation and registered charity guided by the Fundamental Principles of Humanity, Impartiality, Neutrality, Independence, Voluntary Service, Unity and Universality (Canadian Red Cross, 2024a). With more than 2900 staff and 14,000 volunteers, and training more than 850,000 people in first aid in 2022 and 2023, the CRC is known for strong community ties, government relationships, first aid education, and crisis management expertise (Canadian Red Cross, 2023, 2024b, 2024c).

From January 2021 to March 2024, the CRC Opioid Harm Reduction (OHR) program launched a series of new courses and updates to existing courses to train individuals on opioid poisoning response. Courses were offered virtually and in-person, in English and French. Courses were marketed to the public through paid social media ads, posts on CRC and influencers’ social media channels, email campaigns, custom email signatures, a spotlight on the CRC home page, and search engine optimization.

The CRC OHR program added content to existing first aid courses and created three new courses, Becoming an Opioid Harm Reduction Champion (“Champions”), Becoming an Opioid Harm Reduction Leader (“Leaders”), and First Aid for Opioid Poisoning Emergencies (“FAOPE”). Content added to existing first aid courses mainly focused on providing a step-by-step guide of how to respond to opioid poisoning using naloxone. The Champions course focused on spreading awareness of opioid poisoning through social media, while the Leaders course was a train-the-trainer offering designed to prepare individuals to train members of their community on opioid poisoning response. Existing first aid course participants and Champions would be provided with a list of organizations that typically distribute naloxone, but they would not be provided with naloxone through the training. Leaders received naloxone kits to distribute within their community. FAOPE provided more information on the opioid poisoning crisis, strategies to reduce stigma, and risk factors for opioid poisoning than the content added to existing first aid courses. After completing the FAOPE course, participants could choose to receive a naloxone kit in the mail or receive a kit if completing the in-person version of the course. Clinical and technical information regarding opioid poisoning response was similar in all the courses. Further information about the program’s courses can be found in Table 1.

**Table 1:** Background, participant eligibility, course objectives, opioid harm reduction content in each course type, and description of naloxone distribution

Existing first aid courses	
Background	The CPR/AED, Emergency First Aid, Standard First Aid, and Workplace Standard First Aid & CPR/AED are paid courses that provide general first aid knowledge and skills. In March 2021, content on opioid harm reduction (OHR) was added as a separate module in each course
Participant eligibility	Anyone residing in Canada
Course objectives	(1) Learn how to reduce opioid-related stigma (2) Recognize and respond to suspected opioid poisoning (3) Learn how to administer intranasal naloxone
OHR content	• Short background on opioids and their effects on the body • The Canadian opioid poisoning crisis • Stigma related to opioid poisoning and opioid use disorder (e.g. what stigma means, language choices) • How to recognize and respond to opioid poisoning, with a greater focus on naloxone • Canadian laws and regulations
Becoming an Opioid Harm Reduction Champion (Champions) course	
Background	The Becoming an Opioid Harm Reduction Champion course was intended to introduce opioid poisoning response to people without prior knowledge and to encourage sharing harm reduction messages via social media. The free, self-directed course ran from March 2022 to August 2024
Participant eligibility	Individuals 13 years or older residing in Canada
Course objectives	(1) Form an identity as an OHR Champion (a person who shares harm reduction messages through social media) (2) Consider the impact of sharing messages on reducing stigma and responding to opioid poisoning (3) Understand the goals and values of the CRC OHR Program
OHR content	• The Canadian opioid poisoning crisis • Harm reduction principles • Stigma related to opioid poisoning and opioid use disorder • Recognizing opioid poisoning • Activating emergency services • Performing cardiopulmonary resuscitation (CPR) • Administering naloxone • Providing continuous care • Where to get naloxone • How to be an OHR Champion
Becoming an Opioid Harm Reduction Leader (Leaders) course	
Background	The Becoming an Opioid Harm Reduction Leader course was an online, self-directed, free, train-the-trainer course intended to reach a larger audience, particularly in rural and remote areas. It ran from April 2023 to March 2024
Participant eligibility	CRC volunteers and training partners at least 18 years old and previously certified in first aid
Course objectives	(1) Build confidence as an OHR Leader (a CRC volunteer or training partner trained to deliver the First Aid for Opioid Poisoning Emergencies course) (2) Gain skills, knowledge, and resources to deliver OHR courses (3) Learn how to support community members’ learning
OHR content	• Technical details on suggested formats, tools, maximum number of participants, learning options, and other tips for facilitating training • Overview of the First Aid for Opioid Poisoning Emergencies course
First Aid for Opioid Poisoning Emergencies (FAOPE) course	
Background	The last stage of the program centred around the distribution of naloxone kits. In August 2022, an in-person FAOPE course was launched, delivered by CRC staff or OHR Leaders to organizations that requested the training. An online, self-directed, free version ran from October 2022 to August 2024
Participant eligibility	Anyone 16 years or older residing in Canada
Course objectives	(1) Become knowledgeable and confident in how to respond to opioid poisoning (2) Learn about opioids and naloxone and how they affect the body (3) Explore ways to reduce stigma against opioid poisoning and naloxone
OHR content	• Background on opioids, naloxone, the unregulated drug supply, substance use disorders, and the scale of the Canadian opioid poisoning crisis • Harm reduction principles • How to reduce stigma related to opioid poisoning and opioid use disorder • Risks of opioid poisoning and how to prevent it • How to access and care for a naloxone kit • National laws and regulations • How to recognize opioid poisoning • How to administer intramuscular and intranasal naloxone • How to perform cardiopulmonary resuscitation (CPR) and use an automated external defibrillator (AED) • Self-care after responding to an emergency
Naloxone distribution	
Participants who completed the OHR Leaders or the FAOPE courses online from October 2022 to September 2023 could order a free mail-delivered intranasal naloxone kit. Individuals who were trained by OHR Leaders or those who completed an in-person offering of the FAOPE course were provided with a naloxone kit after the training completed. Each kit included 2 doses of naloxone hydrochloride intranasal spray (NARCAN™ Nasal Spray, 4 mg/0.1 ml), 1 rescue breathing barrier, 1 pair of disposable gloves, and 1 handout on responding to opioid poisoning. Kits were mailed to all Canadian provinces except Québec	

### Evaluation methods

The CRC collaborated with the University of Toronto Dalla Lana School of Public Health to evaluate the OHR program using quantitative and qualitative methods and lead collaborations with a community advisory council. The program was designed, continuously adapted, and evaluated in partnership with the OHR Advisory Council, which included people with lived and living experience of substance use and harm reduction community leaders and advocates, including from Indigenous communities. Council members met monthly to discuss program changes and evaluation findings, to ensure CRC’s OHR offerings were reflective of the needs of people who use drugs. Process evaluation reports were provided to the CRC every 6 months, with an outcome evaluation report delivered in March 2024.

### Gap assessment

Guided by a Performance Measurement and Evaluation Plan approved by Health Canada, the evaluation included a gap assessment (available in English and French) and a literature review (dos Santos et al., 2024) on virtual opioid poisoning education and naloxone distribution programs. The gap assessment identified priority areas, populations, and strategies for the CRC OHR program. The recommendations included amplifying existing programs, addressing opioid-related stigma through training, and ensuring underserved populations were appropriately supported and their expertise reflected through community engagement. Identified priority groups included males aged 29–40, Indigenous individuals, those working in the construction industry, individuals experiencing homelessness, those living in British Columbia, Alberta, and Ontario, and people living in rural and remote areas.

### Participants

Evaluation participants were those residing in Canada who could communicate in English or French and who completed a demographic and/or feedback survey administered through the OHR courses. In total, 55,473 people completed the demographic survey and 208,106 the feedback survey. Due to program funding stipulations, the reported number of participants who completed a course excluded Québec. However, demographic and feedback survey data do include participants from Québec.

### Data collection

The program collected data through different methods at different time points (Table 2). Surveys differed slightly between courses due to updates or changes in content for target audiences (see Appendix, Table 6). Data collected from April 2023 onwards is reported here to present data from the same time period for the Champions, Leaders and FAOPE course (which launched in April 2023).

**Table 2:** Method of data collection, time point of data collection, and data collected throughout the program

Method	Time point of data collection	Data collected
Course rosters	Required to be added by a training partner to an internal platform after an in-person first aid course	• Course type • Number of participants • Province/territory of residence • Language of the course
Demographic survey	Required before any online course, except for existing first aid courses	• Age • Gender • Race/ethnicity • Province/territory of residence • Work industry
Feedback survey	Voluntary after any course	• Knowledge of responding to opioid poisoning • Confidence to respond to opioid poisoning • Likelihood of responding to opioid poisoning • How participants learned about the program • Duration to complete the course • Previous training in opioid poisoning • Previous experience witnessing, personally experiencing, or helping in an opioid poisoning • Additional voluntary feedback

### Data analysis and reporting

Survey data were analyzed using R Studio 4.2.1. Exploratory and visual analyses were used to review variable distributions and outcomes. Since participants voluntarily filled out surveys, non-parametric tests were used, which do not assume a normal distribution. The Wilcoxon signed-rank test was used to compare self-reported knowledge and confidence ratings before and after the courses, as well as before and after ratings for people with prior opioid-related training and those without. Prior training included any self-reported education or training regarding opioid poisoning. An alpha of 0.05 was adopted for all tests. Data including fewer than six individuals were suppressed to avoid participants’ re-identification. Quantitative data collected through the online and in-person versions of the FAOPE course were aggregated as the number of respondents to the in-person course feedback survey (*n* = 13) did not allow for meaningful disaggregation.

Qualitative analyses followed Braun and Clarke’s method to analyze 4952 open-ended responses from the online version of the FAOPE course (Braun & Clarke, 2006). This course was selected for thematic analysis due to its critical importance within the program, the high volume of feedback it received, and the general relevance of insights from this program to other courses. Phase 1 involved cleaning the dataset, removing 1890 comments such as “all good”, “no”, and “N/A”. In phase 2, a word cloud was created using NVivo 14 to code the remaining 3062 responses. In phase 3, feedback was categorized into three themes: positive, negative, and constructive. Phase 4 ensured accurate categorization, and Phase 5 assessed each theme for clarity. After individual coding, in Phase 6, reviewers reached a consensus on themes. Since individual comments may cover multiple themes, each section was categorized separately when appropriate. Therefore, we report the number of “references” instead of the number of comments.

## Outcomes

Table 3 reports the number of participants in each course and the number of respondents to the demographic and feedback survey. The program delivered 1,386,995 trainings in opioid harm reduction, 97.1% through existing first aid courses (Table 3).

**Table 3:** Number of people who completed each course type and respondents of the demographic and feedback surveys

Course type	Demographic survey respondents (n, %)	Participants who completed the course (n, %)	Feedback survey respondents (n, %)
Existing first aid courses	N/A	1,346,933 (97.1%)	183,474 (88.2%)
Champions course	9812 (17.7%)	7134 (0.51%)	4877 (2.3%)
Leaders course	171 (0.3%)	190 (0.01%)	65 (0.03%)
FAOPE course	45,490 (82.0%)	32,738 (2.36%)	19,690 (9.5%)
Total	55,473	1,386,995	208,106

### Program participants

The percentage and count for each demographic category by course, as well as information on the duration it took for participants to complete courses, how they learned about it, and the number of individuals receiving opioid poisoning–related training for the first time and people with no experience witnessing, personally experiencing, or responding to opioid poisoning, are presented in Table 4. Compared to the 2021 Canadian Census (Statistics Canada, 2023), the program reached a representative sample of Indigenous individuals (5.3% vs. 4.9%) and rural residents (25.2% vs. 17.8%) (Statistics Canada, 2022). However, it had an underrepresentation of men (34.3% vs. 48.8%) and individuals working in the construction industry (4.8% vs. 7.6%).

**Table 4:** Demographic categories, course duration and dissemination strategy, first-time receiving opioid poisoning–related training**,** and personal experience with opioid poisoning

Variable	Existing first aid courses (%, *n*)	Champions course (%, *n*)	Leaders course (%, *n*)	FAOPE course (%, *n*)	Total (%, *n*)
Gender					
Man	34.5% (*n* = 46,635)	30.2% (*n* = 2979)	25.9% (*n* = 44)	34.7% (*n* = 15,408)	34.3% (*n* = 65,066)
Woman	62.6% (*n* = 84,668)	65.7% (*n* = 6479)	47.6% (*n* = 81)	60.9% (*n* = 27,066)	62.3% (*n* = 118,294)
Non-binary	0.9% (*n* = 1164)	1.4% (*n* = 134)	N/A	1.1% (*n* = 468)	0.9% (*n* = 1766)
Transgender man	0.1% (*n* = 112)	0.3% (*n* = 27)	N/A	0.3% (*n* = 126)	0.1% (*n* = 265)
Transgender woman	0.03% (*n* = 46)	0.1% (*n* = 14)	N/A	0.1% (*n* = 48)	0.1% (*n* = 108)
Two-spirit	0.1% (*n* = 76)	0.3% (*n* = 32)	N/A	0.3% (*n* = 135)	0.1% (*n* = 243)
My identity is not represented here*	0.4% (*n* = 590)	0.5% (*n* = 49)	N/A	0.3% (*n* = 122)	0.4% (*n* = 761)
Prefer not to say	1.5% (*n* = 2062)	1.4% (*n* = 142)	22.9% (*n* = 39)	2.4% (*n* = 1053)	1.7% (*n* = 3296)
Total respondents	135,353	9856	164	44,426	189,799
Province of residence					
Alberta	19.9% (*n* = 161,781)	14.1% (*n* = 1388)	15.9% (*n* = 27)	12.9% (*n* = 5753)	19.5% (*n* = 168,949)
British Columbia	13.4% (*n* = 108,457)	14.5% (*n* = 1429)	11.8% (*n* = 20)	11.8% (*n* = 5254)	13.3% (*n* = 115,160)
Manitoba	4.9% (*n* = 39,673)	5.2% (*n* = 509)	7.6% (*n* = 13)	5.3% (*n* = 2369)	4.9% (*n* = 42,564)
New Brunswick	2.6% (*n* = 20,956)	2.8% (*n* = 277)	N/A	3.2% (*n* = 1402)	2.6% (*n* = 22,635)
Newfoundland and Labrador	2.2% (*n* = 17,891)	5.0% (*n* = 491)	3.5% (*n* = 6)	8.7% (*n* = 3863)	2.6% (*n* = 22,251)
Northwest Territories	0.1% (*n* = 915)	0.2% (*n* = 18)	N/A	0.2% (*n* = 88)	0.1% (*n* = 1021)
Nova Scotia	2.2% (*n* = 17,903)	3.4% (*n* = 335)	4.1% (*n* = 7)	3.1% (*n* = 1378)	2.3% (*n* = 19,623)
Nunavut	0.1% (*n* = 613)	N/A	N/A	0.1% (*n* = 30)	0.1% (*n* = 643)
Ontario	49.3% (*n* = 399,901)	45.5% (*n* = 4472)	37.1% (*n* = 63)	46.1% (*n* = 20,502)	49.1% (*n* = 424,938)
Prince Edward Island	0.4% (*n* = 3078)	0.5% (*n* = 46)	N/A	0.6% (*n* = 245)	0.4% (*n* = 3369)
Quebec	N/A	3.2% (*n* = 319)	N/A	1.8% (*n* = 788)	0.1% (*n* = 1107)
Saskatchewan	4.7% (*n* = 37,890)	4.1% (*n* = 400)	9.4% (*n* = 16)	4.6% (*n* = 2036)	4.7% (*n* = 40,342)
Yukon	0.2% (*n* = 1928)	0.2% (*n* = 19)	N/A	0.2% (*n* = 77)	0.2% (*n* = 2024)
Prefer not to say	N/A	1.3% (*n* = 128)	N/A	1.4% (*n* = 641)	0.1% (*n* = 769)
Total respondents	810,986	9831	152	44,426	865,395
Age group					
* ≤ 24	N/A	13–24: 22.6% (*n* = 2227)	18–24: 16.5% (*n* = 28)	15–24: 20.4% (*n* = 9124)	13–24: 20.8% (*n* = 11,379)
25–34	N/A	24.9% (*n* = 2451)	13.5% (*n* = 23)	24.1% (*n* = 10,782)	24.2% (*n* = 13,256)
35–44	N/A	22.3% (*n* = 2201)	19.4% (*n* = 33)	22.5% (*n* = 10,046)	22.4% (*n* = 12,280)
45–54	N/A	16.5% (*n* = 1624)	15.3% (*n* = 26)	17.5% (*n* = 7830)	17.3% (*n* = 9480)
55–64	N/A	9.3% (*n* = 914)	8.8% (*n* = 15)	10.5% (*n* = 4704)	10.3% (*n* = 5633)
≥ 65	N/A	2.7% (*n* = 268)	5.3% (*n* = 9)	2.6% (*n* = 1158)	2.6% (*n* = 1435)
Prefer not to say	N/A	1.7% (*n* = 171)	21.2% (*n* = 36)	2.3% (*n* = 1037)	2.3% (*n* = 1244)
Total respondents	N/A	9856	170	44,681	54,707
Race/ethnicity					
Black	N/A	16.7% (*n* = 855)	7.6% (*n* = 13)	11.6% (*n* = 3041)	12.4% (*n* = 3909)
East Asian	N/A	3.1% (*n* = 160)	N/A	3.1% (*n* = 808)	3.1% (*n* = 969)
Indigenous	N/A	5.9% (*n* = 301)	4.7% (*n* = 8)	5.2% (*n* = 1355)	5.3% (*n* = 1664)
Latin American	N/A	2.0% (*n* = 103)	N/A	1.9% (*n* = 491)	1.9% (*n* = 595)
Middle Eastern	N/A	3.0% (*n* = 153)	N/A	2.8% (*n* = 738)	2.8% (*n* = 895)
South Asian	N/A	15.4% (*n* = 789)	4.1% (*n* = 7)	9.9% (*n* = 2579)	10.7% (*n* = 3375)
Southeast Asian	N/A	4.4% (*n* = 224)	N/A	4.3% (*n* = 1129)	4.3% (*n* = 1357)
White	N/A	41.9% (*n* = 2142)	49.4% (*n* = 84)	52.5% (*n* = 13,727)	50.8% (*n* = 15,953)
Another race category**	N/A	0.8% (*n* = 42)	N/A	0.8% (*n* = 199)	0.8% (*n* = 241)
Mixed race	N/A	2.6% (*n* = 135)	3.5% (*n* = 6)	3.0% (*n* = 775)	2.9% (*n* = 916)
Do not know	N/A	0.7% (*n* = 35)	N/A	0.7% (*n* = 178)	0.7% (*n* = 215)
Prefer not to say	N/A	3.4% (*n* = 172)	23.5% (*n* = 40)	4.3% (*n* = 1122)	4.2% (*n* = 1334)
Total respondents	N/A	5111	170	26,142	31,423
Area of residence					
Remote	N/A	2.9% (*n* = 287)	N/A	2.6% (*n* = 1137)	2.6% (*n* = 1429)
Rural	N/A	23.6% (*n* = 2325)	20.0% (*n* = 34)	25.6% (*n* = 11,380)	25.2% (*n* = 13,739)
Suburban	N/A	20.3% (*n* = 2001)	14.1% (*n* = 24)	22.1% (*n* = 9823)	21.8% (*n* = 11,848)
Urban	N/A	47.0% (*n* = 4637)	42.4% (*n* = 72)	43.2% (*n* = 19,201)	43.9% (*n* = 23,910)
Prefer not to say	N/A	6.1% (*n* = 606)	20.6% (*n* = 35)	6.5% (*n* = 2885)	6.5% (*n* = 3526)
Total respondents	N/A	8449	170	44,426	54,452
Work industry					
Agriculture	N/A	0.5% (*n* = 53)	2.1% (*n* = 6)	1.5% (*n* = 844)	1.3% (*n* = 903)
Construction	N/A	3.2% (*n* = 320)	6.2% (*n* = 18)	5.1% (*n* = 2936)	4.8% (*n* = 3274)
Education	N/A	10.4% (*n* = 1027)	16.5% (*n* = 48)	10.0% (*n* = 5752)	10.1% (*n* = 6827)
Finance	N/A	0.9% (*n* = 92)	N/A	1.2% (*n* = 708)	1.2% (*n* = 803)
Fishing	N/A	0.2% (*n* = 19)	N/A	0.7% (*n* = 425)	0.7% (*n* = 449)
Forestry	N/A	0.3% (*n* = 28)	N/A	0.7% (*n* = 423)	0.7% (*n* = 455)
Government	N/A	5.6% (*n* = 549)	6.5% (*n* = 19)	7.5% (*n* = 4301)	7.2% (*n* = 4869)
Healthcare	N/A	30.0% (*n* = 2954)	17.2% (*n* = 50)	23.9% (*n* = 13,785)	24.7% (*n* = 16,789)
Hospitality	N/A	4.1% (*n* = 403)	3.4% (*n* = 10)	5.9% (*n* = 3427)	5.7% (*n* = 3840)
Manufacturing	N/A	1.7% (*n* = 165)	N/A	2.9% (*n* = 1680)	2.7% (*n* = 1849)
Mining and energy	N/A	0.8% (*n* = 77)	2.1% (*n* = 6)	1.5% (*n* = 843)	1.4% (*n* = 926)
Non-profit	N/A	12.9% (*n* = 1267)	14.4% (*n* = 42)	10.8% (*n* = 6255)	11.1% (*n* = 7564)
Retail services	N/A	3.1% (*n* = 302)	3.1% (*n* = 9)	6.9% (*n* = 3996)	6.3% (*n* = 4307)
Technology	N/A	1.6% (*n* = 156)	3.1% (*n* = 9)	2.2% (*n* = 1242)	2.1% (*n* = 1407)
Transportation	N/A	1.4% (*n* = 138)	N/A	3.3% (*n* = 1902)	3.0% (*n* = 2045)
None of the above	N/A	16.8% (*n* = 1659)	5.2% (*n* = 15)	11.2% (*n* = 6485)	12.0% (*n* = 8159)
Prefer not to say	N/A	6.6% (*n* = 647)	13.1% (*n* = 38)	4.7% (*n* = 2708)	5.0% (*n* = 3393)
Total respondents	N/A	9856	291	57,712	67,859
Course language					
English	98.8% (*n* = 1,260,906)	97.1% (*n* = 9573)	96.5% (*n* = 164)	98.0% (*n* = 43,782)	98.8% (*n* = 1,314,425)
French	1.2% (*n* = 15,147)	2.9% (*n* = 283)	3.5% (*n* = 6)	2.0% (*n* = 901)	1.2% (*n* = 16,337)
Total respondents	1,276,053	9856	170	44,683	1,330,762
Course information					
Duration to complete the course	N/A	20–30 min (45.5%, *n* = 2209)	N/A	60 min (median)	N/A
Main strategy participants learned about the course	N/A	Red Cross website (28.6%, *n* = 817)	A Red Cross Training Partner (29.5%, *n* = 23)	A friend, colleague, or family member (27.9%, *n* = 5951)	N/A
First time receiving opioid poisoning–related training and no personal experience with opioid poisoning					
First-time learners	N/A	38.9% (*n* = 986)	11.3% (*n* = 7)	49.5% (*n* = 6349)	7342
No personal experience	N/A	64.8% (*n* = 1638)	51.6% (*n* = 33)	73.5% (*n* = 9422)	11,093

*Participants who chose “My identity is not represented here” for gender options reported being agender, bigender, demiboy/girl, gender non-conforming, genderdynamic, genderqueer, intersex, multigender, pangender, pomogender, queer, questioning, transqueer, all categories, or none

**Participants who chose “Another race category” reported being African, African-Arab, Arab, Caribbean, Caucasian, Coloured, East Indian, Filipino, Guyanese, Haitian, Indian, Mennonite, Nigerian, Pacific Islander, West Indian, and White Canadian

### Knowledge, confidence, and likelihood of responding to opioid poisoning

**Fig. 1:**
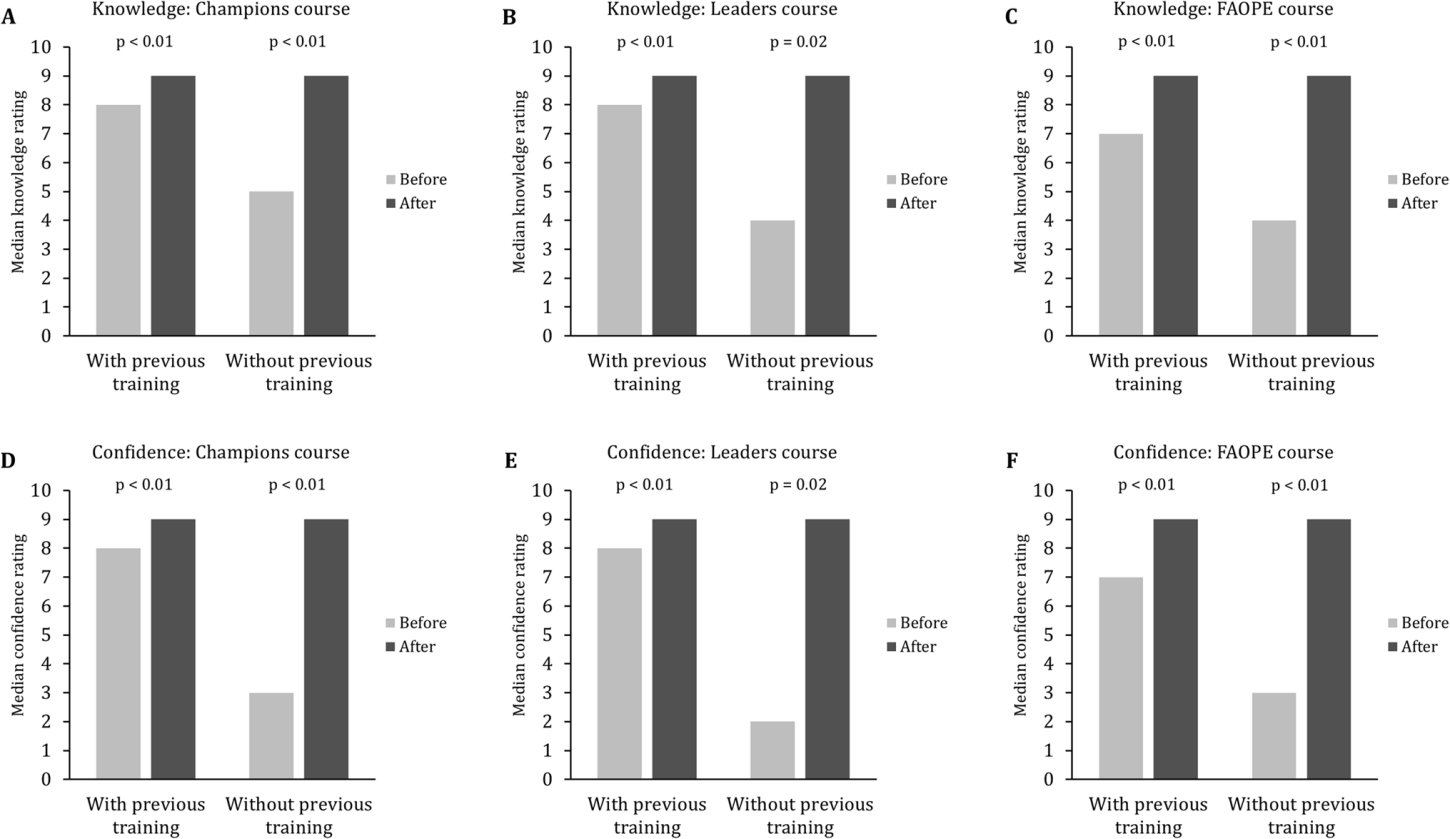
Median knowledge and confidence self-reported ratings by participants’ previous training experience

There was an increase in median self-reported ratings of knowledge of opioid poisoning on a 0 to 10 Likert scale for participants of the Champions (*n* = 1543 with and *n* = 986 without previous training), Leaders (*n* = 55 with and *n* = 7 without previous training), and FAOPE (*n* = 6474 with and *n* = 6358 without previous training) courses (Fig. 1). Similarly, there was an increase in median self-reported ratings of confidence in responding to an opioid poisoning on a 0 to 10 Likert scale for participants of all courses.

Overall, 98.1% of Champions, Leaders, and FAOPE course participants (*n* = 13,754) self-reported that they were more likely to help someone experiencing opioid poisoning after their training.

### Naloxone distribution

From October 2022 to March 2024, participants who completed the Leaders (33%) or the FAOPE (67%) courses could receive a naloxone kit by mail or provided to them in-person. The OHR program distributed 24,098 intranasal naloxone kits. Most kits (75.1%) were delivered to residents of Ontario (33.2%), Alberta (14.7%), Manitoba (14.6%), and British Columbia (12.6%).

### Qualitative feedback

A total of 3826 references were coded from the 3062 comments about the online version of the FAOPE course (Table 5). References were categorized into positive feedback (82%, *n* = 3126), indicating satisfaction; constructive feedback (13%, *n* = 487), highlighting areas for improvement; and negative feedback (6%, *n* = 213), concerns with the course.

**Table 5:** Representative comments from the qualitative feedback given by participants of the online FAOPE course

Theme	Representative comments^a^
Positive feedback	“Excellent course! Took a holistic approach to remove stigma and provide support.”
	“Great course. I really appreciate being able to learn and build my confidence so I can help in my community. I hadn’t even heard of intranasal naloxone before so it’s great to know about the different options.”
	“Thank you for making this training accessible. Learning about the Good Samaritan law and knowing I can’t harm someone using Naloxone makes a big difference in my willingness to help.”
Constructive feedback	“[…] Avoiding gray text and always using black on white or white on black makes the text clearer for all. Options to personalize colour scheme, text sizes, and fonts within the platform could also enhance access.”
	“Give a tutorial on how to use both nasal and intramuscular naloxone because different places give out different types.”
	“I would like to know more about what kind of care people need after the 20–90-min naloxone efficacy. Just in case they can’t access medical care for long, or even to help them know what will be probably happening next. Or also to know about safe and respectful ways to make boundaries with bystanders, if there are other people using substances nearby or interacting with the scene.”
Negative feedback	“It was a little too easy – but I have also done a lot of first aid training so maybe that is why!”
	“This course was very dumbed down and way longer than needed. I also feel that this course focused more on reducing stigma than was needed for a CRC course—that made it seem less medical and more political, which is NOT a good thing coming from a medical course.”
	“[…] I disagree with a lot of the advocacy content around things like person-first language or the medicalizing of substance use. I appreciate the approach that was taken to talk about destigmatizing addiction but it’s certainly not the same one taken by all Canadian advocates.”

^a^Basic punctuation and grammar have been corrected for clarity

Participants commented that the course increased their confidence to recognize opioid poisoning and administer naloxone (*n* = 686). Respondents appreciated the simplicity and conciseness of the course (*n* = 144), with particular mention of the range of topics covered, and the beneficial use of videos. Participants also appreciated the content on stigma, including person-first language and the use of the term “poisoning” rather than overdose (*n* = 42). Other reported positive aspects included that the course was free, was delivered online, and could be completed in under an hour (*n* = 43)*.* Constructive feedback included requests for more information on intramuscular naloxone, as the course initially only discussed the intranasal format (*n* = 106). Participants recommended to include audio and closed captions in videos, audible reader capability for the online course platform, to use accessible colour schemes, fonts, and text sizes, and to incorporate more images and videos (*n* = 91). The use of the words “illegal opioids” in the course was criticized (*n* = 27) and it was suggested to replace these with “unregulated opioids”. Negative feedback included disagreements on person-first language (*n* = 18), justifying that it can be problematic to some communities.

## Discussion

The CRC OHR program delivered 1,386,995 trainings and distributed 24,098 intranasal naloxone kits. Participants of OHR courses self-reported increased ratings of knowledge of opioid poisoning and confidence in responding to opioid poisoning, particularly among those without prior training. The program was free for participants, bilingual, and delivered virtually and in-person. Further, the program was developed and continuously updated considering the feedback from course participants and people with living or lived experience of drug use. The program successfully reached target populations including Indigenous communities, rural residents, and individuals with no training in opioid poisoning response. Moreover, intranasal naloxone kits were distributed to the three provinces with the highest rates of opioid-related fatalities (Federal, provincial, and territorial Special Advisory Committee on Toxic Drug Poisonings, 2024). The mail-delivery system further ensured accessibility across Canada, addressing location and availability barriers (Bardwell & Lappalainen, 2021; dos Santos et al., 2024).

Despite these successes, the program did not measure participant changes to knowledge and confidence through validated scales, observe actionable skills rather than self-reported knowledge, or measure long-term trends on participants’ knowledge and confidence to respond to opioid poisoning. The program’s reach among men and those who work in the construction industry—some of the groups most affected by opioid poisoning fatalities (Federal, provincial, and territorial Special Advisory Committee on Toxic Drug Poisonings, 2024; Gomes et al., 2022)—was limited. Social expectations about gender often delay these groups’ access to healthcare (CATIE, 2023; Collins et al., 2019). Future versions of the program could benefit from customizing language and content and revisiting recruitment strategies to better resonate and engage with these populations (CATIE, 2023; Collins et al., 2019). Though the program reached rural and Indigenous participants, a merely representative number may be insufficient for meaningful engagement, and future programs may set more ambitious goals given the disproportionate impact of the opioid poisoning crisis on these and other populations experiencing marginalization.

## Conclusion

While opioid poisoning education and naloxone distribution programs have conventionally been delivered by healthcare and community-based agencies, this program engaged a national humanitarian organization; a first-of-its-kind initiative in Canada. Humanitarian organizations can advance harm reduction and other services and amplify efforts to address the opioid poisoning crisis through their expansive reach and community ties. Humanitarian organizations like the Canadian Red Cross are uniquely positioned to integrate opioid harm reduction into their broader public health crisis response initiatives, thereby expanding the dissemination and impact of life-saving interventions.

## Implications for policy and practice

What are the innovations in this program?

- Collaboration with people with lived experiences of drug use ensured the content was relevant, respectful, and effective in addressing the specific challenges faced by this population.
- The program’s courses were designed to be accessible and adaptable to various populations, including laypersons from all ages, and those in rural and remote areas.
- The free naloxone distribution, particularly through mail delivery, was a critical innovation in making life-saving tools available nationwide.

What are the burning research questions for this innovation?

- What are the most effective strategies for customizing opioid harm reduction training to better engage high-risk groups such as men and individuals who work in the construction industry?
- Were the naloxone kits distributed through the CRC OHR program successfully used to reverse opioid poisonings across Canada?
- What are the sustained impacts on participants’ knowledge, attitudes, and behaviours related to opioid poisoning and naloxone use after completing the CRC OHR program?

## Data Availability

Data are not publicly available and are protected under a data sharing agreement between the Canadian Red Cross Society and the University of Toronto.

## Appendix

**Table 6:** Feedback survey questions by course and period

**Existing first aid courses**	**Champions course**		**Leaders course**	**First Aid for Opioid Poisoning Emergencies course**	
	**March 2022–March 2023**	**April 2023–March 2024**		**Online**	In-person
This course made me more likely to stop and help — in any way — a stranger who needs help.^a^					
Is there any additional feedback you would like to share?^b^					
		How did you hear about the course?^c^			
	How long did you take to complete the online course?^d^			How long did you take to complete the online course?^d^	
		How knowledgeable did you feel about opioid poisoning before/after taking this course?^e^			
		How confident do you feel about opioid poisoning before/after taking this course?^e^			
		Before taking this course, have you witnessed, personally experienced, or responded to opioid poisoning?^f^			
		Before taking this course, have you received any opioid poisoning-related education/training?^f^			

^a^Likert scale (strongly disagree, disagree, agree, strongly agree)

^b^Open-ended

^c^Check all that apply: (a) A friend, colleague, or family member, (b) A Red Cross Training Partner, (c) A Red Cross Instructor (while attending other training), (d) Red Cross website, (e) Internet search, (f) Social media, (g) Other (please specify)

^d^Select one: (a) 10 min or less, (b) 11–20 min, (c) 20–30 min, (d) More than 30 min

^e^Numerical scale from 0 to 10, with 0 being “not at all …” and 10 being “extremely …”

^f^Select one: a) Yes, b) No

## Code availability

Code is not publicly available and is protected under a data sharing agreement between the Canadian Red Cross Society and the University of Toronto.
